# Bacterial-Mediated In Situ Engineering of Tumour-Associated Macrophages for Cancer Immunotherapy

**DOI:** 10.3390/cancers17050723

**Published:** 2025-02-20

**Authors:** Gabriela Christina Kuhl, Mark Tangney

**Affiliations:** 1 Cancer Research @UCC, College of Medicine and Health, University College Cork, T12 K8AF Cork, Ireland; gkuhl@ucc.ie; 2 APC Microbiome Ireland, University College Cork, T12 YT20 Cork, Ireland

**Keywords:** recombinant microbes, gene and cell therapy, drug delivery systems

## Abstract

Background/Objectives: Tumour-associated macrophages (TAMs) are critical components of the tumour microenvironment (TME), significantly influencing cancer progression and treatment resistance. This review aims to explore the innovative use of engineered bacteria to reprogram TAMs, enhancing their anti-tumour functions and improving therapeutic outcomes. Methods: We conducted a systematic review following a predefined protocol. Multiple databases were searched to identify relevant studies on TAMs, their phenotypic plasticity, and the use of engineered bacteria for reprogramming. Inclusion and exclusion criteria were applied to select studies, and data were extracted using standardised forms. Data synthesis was performed to summarise the findings, focusing on the mechanisms and therapeutic benefits of using non-pathogenic bacteria to modify TAMs. Results: The review summarises the findings that engineered bacteria can selectively target TAMs, promoting a shift from the tumour-promoting M2 phenotype to the tumour-fighting M1 phenotype. This reprogramming enhances pro-inflammatory responses and anti-tumour activity within the TME. Evidence from various studies indicates significant tumour regression and improved immune responses following bacterial therapy. Conclusions: Reprogramming TAMs using engineered bacteria presents a promising strategy for cancer therapy. This approach leverages the natural targeting abilities of bacteria to modify TAMs directly within the tumour, potentially improving patient outcomes and offering new insights into immune-based cancer treatments. Further research is needed to optimise these methods and assess their clinical applicability.

## Simple Summary

This research focuses on tumour-associated macrophages (TAMs), which are immune cells that play a crucial role in cancer development. The authors aim to understand how these cells can be reprogrammed to fight cancer more effectively. By using specially engineered bacteria, they hope to target and modify TAMs directly within the tumour, enhancing their ability to attack cancer cells. This innovative approach could lead to more effective cancer treatments by harnessing the body’s own immune system. The findings from this research may provide new strategies for cancer therapy, potentially improving outcomes for patients and offering valuable insights for the research community.

## 1. Introduction

Tumour-associated macrophages (TAMs) are among the most abundant immune cells within the tumour microenvironment (TME) and can represent more than 50% of tumour-infiltrating immune cells [1]. TAMs dynamically influence the cancer ecosystem evolution, composing a complex scenery in tumour biology [2]. The presence of TAMs has become a hallmark of cancer malignancy, with pro-tumoural functions ranging from promoting tumour growth to immune suppression and treatment resistance [3]. Emerging evidence suggests that TAMs are involved in tumour progression via multiple mechanisms [4]. During the tumour initiation phase, macrophages can directly contribute to tumour-promoting inflammation by targeting malignant cell death by phagocytosis, or indirectly by the secretion of proinflammatory cytokines [5,6]. Depending on their activation status, macrophages can promote tumour progression, induce angiogenesis and metastasis, and contribute to tumour invasion and resistance to therapy [7]. TAM-derived products also serve as signalling mediators, influencing the behaviour of adjacent cells [8]. Existence of tissue resident and recruited TAMs further adds to the complexity of metabolic pathways in various macrophage subsets [9]. These processes occur in concert with tumour, immune, or stromal cell adaptation in the TME, generating an interconnected system that drives cancer progression and evolution [10].

As immune cells within the TME, TAMs are a heterogeneous group of macrophages that respond to various factors and can be polarised into phenotypes with opposing activity. Macrophage polarisation refers to the process by which macrophages are activated into specific and varied functional profiles. The functional states of macrophages are generally divided into two main types: M1, which are classically activated, and M2, which are alternatively activated [11]. The M1 phenotype is associated with pro-inflammatory and anti-tumour functions, while the M2 phenotype supports anti-inflammatory responses and can promote tumour growth. These divergent roles are influenced by various factors present in the surrounding microenvironment [12].

Macrophage polarisation oscillates between pro-inflammatory and anti-inflammatory states as a dynamic transition modulated by the cytokine milieu. This phenotypic plasticity is reflective of the macrophage’s responsiveness to distinct cytokine signatures, which dictate their functional orientation within the immune landscape [13,14]. A variety of signalling pathways, along with transcriptional and post-transcriptional mechanisms, govern the expression of sequence-specific transcription factors. These factors in turn define the phenotypes and functional characteristics of macrophages [15,16]. Increasing evidence suggests that recognition receptors, cytokines, and the associated signalling and genetic mechanisms govern the entirety of cell activation [17].

To shift macrophages from a tumour-promoting M2 phenotype to a tumour-fighting M1 phenotype, current approaches employ inhibitors that target the cytokines and chemokines responsible for recruiting and polarising myeloid cells that support tumours [18]. Additionally, activators are used to bolster their anti-tumorigenic and immune-activating capabilities [19]. A notable advance in cancer therapy involves macrophage reprogramming, which has led to tumour regression [8]. This underscores the importance of acknowledging a more extensive range of functions for macrophages beyond the traditional framework.

This review explores how the reprogramming of TAMs influences the regulation of the innate inflammatory response. It investigates the signalling pathways, genetic markers, and functional attributes that macrophages acquire as they become activated. In this context, this article explores the cutting-edge application of engineered bacteria as a macrophage programming technology for various immune therapeutic strategies. Bacteria have the potential to provide significant clinical advantages for broad manipulation of the intratumoural immune system via the simultaneous delivery of DNA and/or proteins.

## 2. Overview of Macrophage Polarisation

Distinct patterns of cytokine secretion are instrumental in shaping the phenotypic and functional attributes of macrophages, delineating their specific roles in reaction to Th1 and Th2 cells [20,21,22]. M1 macrophages are known to elicit Th1 immune responses, while M2 macrophages facilitate Th2 immune reactions [23,24]. The cytokines released by M1 macrophages serve to suppress the growth of nearby cells and can cause damage to adjacent tissues, contributing to anti-tumour responses [12,25]. Conversely, M2 macrophages engage in a series of interconnected, location-specific reactions that result in the release of cytokines, which support the growth of neighbouring cells and aid in tissue repair, aligning with pro-tumour activities [26].

Signal transducer and activator of transcription (STAT) proteins are particularly important in mediating the signals that determine whether a macrophage will adopt an M1 or M2 phenotype. The balance between these STAT proteins and their pathways is essential for the proper functioning of the immune response [27]. The interaction with cytokines from T-cells, particularly the distinct activation pathways of STAT1 and the synergistic effects of STAT3/STAT6, orchestrates the polarisation process and their subsequent functions [28].

STAT1, along with NFκB, is instrumental in M1 polarisation, which is triggered by granulocyte–macrophage colony-stimulating factor (GM-CSF). When activated by interferon-γ (IFNγ), alone or with microbial components like lipopolysaccharides (LPS) or cytokines such as tumour necrosis factor (TNF-α), there is an upsurge in pro-inflammatory cytokines (e.g., IL-1β, IL-2, IL-6, IL-12, IL-23) and a reduction in IL-10 [24,29,30,31]. This results in cytotoxic and tissue-damaging inflammatory actions. Additionally, M1 macrophages are characterised by high levels of antigen presentation, elevated production of nitric oxide (NO) and reactive oxygen intermediates (ROIs). They also typically show increased COX2 and transcription factor IRF5, express co-stimulatory molecules CD80 and CD86, and produce chemokines like CXCL9, CXCL10, and CXCL11 [32].

**Figure 1:**
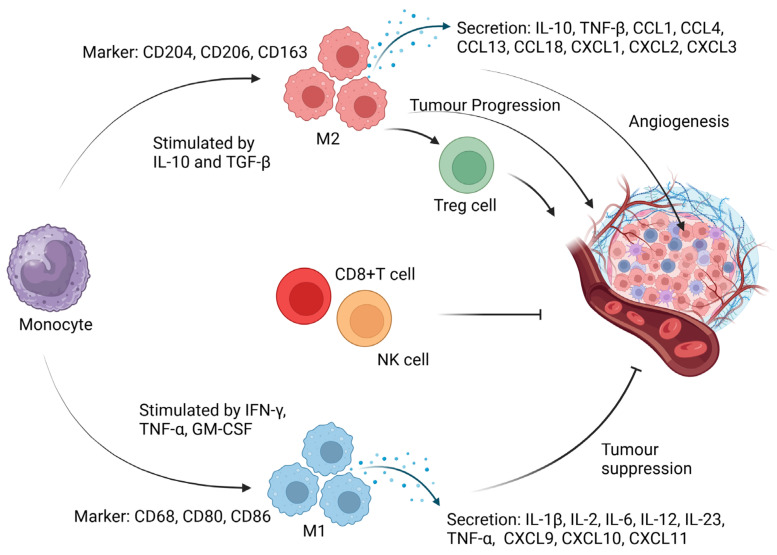
Tumour progression and angiogenesis interaction. TAM serves as a pivotal regulator within the tumour microenvironment, exerting influence over a variety of cellular pathways and interactions. Monocyte: Positioned proximal to the TAM, the monocyte is modulated by cytokines which are critical for its differentiation and function. Cellular Pathways: Treg Cell Pathway: This trajectory culminates in a regulatory T cell (Treg cell), which is intricately linked to an immune suppressor cell, playing a role in immune evasion by the tumour. Angiogenesis Pathway: Depicts the process of new blood vessel formation, a hallmark of tumour progression, facilitated by factors secreted by TAMs. Markers and Cytokines: The diagram includes an array of markers such as CD204 and CD206, characteristic of M2 macrophages, and cytokines like IL-10 and TGF-β, which are indicative of the immunosuppressive environment.

On the other hand, monocytes can differentiate into M2 macrophages under the influence of macrophage colony-stimulating factor (M-CSF) [33]. This transformation is further supported by anti-inflammatory cytokines IL-4 and IL-13, which promote M2 polarisation via STAT6 activation. This activation promotes the expression of genes that support tissue repair, resolution of inflammation, and anti-inflammatory responses [34]. Alternatively, STAT3 activation by IL-10 and IL-6 can prompt the expression of M2 macrophage markers linked to immunosuppression [35]. M2 macrophages are characterised by the upregulation of genes for transforming growth factor-β (TGF-β), cMAF, interferon regulatory factor 4 (IRF4), IL-6, and a variety of chemokines such as CCL1, CCL4, CCL13, CCL18, CXCL1, CXCL2, and CXCL3 [33]. Their capacity to suppress cytotoxic T-cells (CD8+ T-cells) has garnered significant research interest due to its importance in aiding tumour endurance, highlighting potential for clinical applications [36]. Figure 1 illustrates the complex interactions and pathways involving TAMs, including their role in tumour progression and angiogenesis.

The polarisation of macrophages can be a reversible process, led by a switch between pro- and anti-inflammatory cytokine profiles [37,38,39,40,41,42]. Increasing evidence suggests a role of metabolic reprogramming in the regulation of the innate inflammatory response [15,43,44,45]. The modulation of metabolic functions from M2 protumour activities to M1 antitumour responses allows macrophages to respond with appropriate functions in distinct contexts. Strategies aiming repolarisation from the M2 to the M1 phenotype macrophages include inhibitors of cytokines and chemokines involved in the recruitment and polarisation of tumour-promoting myeloid cells, as well as activators of their antitumorigenic and immunostimulant functions [46]. Therefore, the macrophage activation state is functionally distinct and is a key modulator of the host immune response to tumours, key to both tumour development and immunotherapy success or failure [12]. Macrophage-targeting strategies thus provide a possibility to induce a tumour-suppressive effect in cancer immunotherapy [47].

## 3. Macrophage Programming Strategies

Several immunotherapies specifically target macrophages to enhance anti-tumour responses [48]. Reprogramming TAMs significantly influences the regulation of the innate inflammatory response, primarily through their plasticity and ability to switch between pro-inflammatory (M1) and anti-inflammatory (M2) phenotypes [49]. Researchers have developed various techniques to manipulate monocytes and macrophages, which are crucial for enhancing immunotherapy responses [50,51]. A subset of the current strategies includes CSF1R inhibitors, checkpoint inhibitors, antibody–drug conjugates (ADCs), oncolytic viruses, TAM receptor inhibition, nanoparticle-based delivery systems, epigenetic modulation, cytokine therapy, physical and mechanical stimuli, and microbiome-enhanced immunomodulatory therapy [51]. Each approach aims to reprogram macrophages to overcome their immunosuppressive functions and bolster the body’s natural immune response against tumours [52]. The following sections detail some of the key strategies currently being explored, each with its unique mechanism of action and therapeutic potential.

CSF1R Inhibitors: CSF1R inhibitors are used to deplete TAMs, which are often immunosuppressive, thereby enhancing the anti-tumour immune response [48]. Inhibiting CSF1R not only reduces the number of TAMs, but also reprograms the remaining ones to enhance antigen presentation and boost T-cell activation within the tumour environment. This leads to decreased immune suppression and increased interferon responses, effectively restraining tumour progression [53]. Additionally, administering daily doses of a CSF1R inhibitor after radiation therapy significantly lowed the chances of glioblastoma recurrence in mice, with treated mice living more than twice as long as those that were untreated [54].

Checkpoint Inhibitors: Checkpoint inhibitors block proteins used by cancer cells to avoid being attacked by the immune system. These proteins, such as PD-1, PD-L1, and CTLA-4, act as brakes on the immune system. By inhibiting these checkpoints, the drugs release the brakes, allowing T cells to attack cancer cells more effectively [55,56]. The interaction between macrophages and immune checkpoints is a significant area of research, as it holds the potential to enhance the effectiveness of cancer immunotherapies. Blocking these checkpoints can alter macrophage behaviours, potentially leading to improved patient outcomes [57,58,59]. Research from Johns Hopkins found that a novel therapy reprogrammed immune-suppressing macrophages into immune-boosting macrophages, which helped shrink prostate and bladder tumours in mice by blocking the use of glutamine [60].

Antibody–Drug Conjugates (ADCs): ADCs can be designed to target macrophages within the tumour microenvironment, delivering cytotoxic agents directly to these cells to reduce their pro-tumour activities [61]. Interactions between ADCs and TAMs significantly enhance the antitumor activities of ADCs, making them more effective [61]. Recent advancements in ADC design have led to a rapid increase in the number of approved agents for both haematological cancers and solid tumours [62].

Oncolytic Viruses: Oncolytic viruses selectively infect and kill tumour cells and can also modulate the TME by affecting macrophage function [63]. Oncolytic viruses have the remarkable ability to reprogram the TME by increasing the infiltration of M1-like macrophages and T cells into the tumour, repolarising M2-like macrophages, and controlling tumour progression [64]. Additionally, these viruses can disrupt immune-blocking proteins, thereby enhancing the immune response against cancer cells [65].

TAM Receptor Inhibition: TAM receptor signalling plays a significant role in the autonomous oncogenic activity within tumour cells [66]. TAM receptor inhibitors target receptors on TAMs, such as Tyro3, Axl, and MerTK, which help maintain an immunosuppressive environment within tumours. Inhibiting these receptors can reprogram TAMs from an M2 (tumour-promoting) to an M1 (tumour-fighting) state, thereby enhancing the immune response against the tumour [67,68]. Recent literature reviews emphasise TAMs’ critical role in creating an immunosuppressive environment within tumours, either through direct interaction with cancer cells or by modulating the TME to impair T-cell function via immunosuppressive factors [69,70,71,72]. This dual approach of targeting TAM receptors and reprogramming macrophages holds significant promise for improving cancer treatment outcomes.

Nanoparticle-Based Delivery Systems: Nanoparticles can be engineered to deliver drugs directly to tumour-associated macrophages (TAMs). These drugs include Toll-like receptor (TLR) agonists like resiquimod (R848), SHP2 inhibitors such as SHP099, photosensitisers like IR820, chemotherapeutic agents such as doxorubicin, cytokines like IFN-γ, and various small molecule inhibitors that regulate M1 polarisation [73,74,75,76]. By delivering these drugs, the nanoparticles effectively repolarise TAMs from an M2 (pro-tumour) to an M1 (anti-tumour) phenotype [73]. This repolarisation restores the macrophages’ phagocytic activity, enhancing their ability to attack and destroy tumour cells. Additionally, nanostructured drug delivery systems in immunotherapy have shown great potential by increasing drug accumulation at the desired site, thereby improving treatment efficiency and therapeutic outcomes [77].

Epigenetic Modulation: Epigenetic drugs can alter the gene expression profiles of macrophages, promoting a shift from an M2 to an M1 phenotype [78]. This reprogramming enhances their inflammatory and anti-tumour functions, suggesting significant potential applications in cancer therapy [79]. Additionally, epigenetic modulation plays a crucial role in antitumor immunity by promoting transcriptional and metabolic reprogramming in immune cells, leading to improved cancer immunotherapy outcomes [80].

Cytokine Therapy: Administering specific cytokines can modulate macrophage activity, enhancing their anti-tumour functions [81]. Cytokine therapy boosts the immune response against tumours by promoting the M1 phenotype and reducing immune evasion by cancer cells [82]. The clinical application of cytokines in cancer immunotherapy has shown significant therapeutic efficacy, highlighting their potential to improve treatment outcomes and offer new hope for patients [83].

Physical and Mechanical Stimuli: Applying physical stimuli, such as mechanical cues or substrate topography, can significantly influence macrophage differentiation and function [84]. Examples of mechanical cues include fluid shear stress, compression, tension, and hydrostatic pressure [85]. Substrate topography examples include microgrooves, nanopillars, ridges and pits, and stiffness gradients [86]. These stimuli often promote the M1 phenotype, enhancing their pro-inflammatory and anti-tumour activities [87,88]. The importance of mechanical stimulation is also highlighted in organ-on-chip models, where biomechanical cues can drastically alter cellular responses and improve drug development outcomes [89].

Microbiome-Enhanced Immunomodulatory Therapy: Recent advancements in cancer immunotherapy have explored using gut bacteria to distally reprogram macrophages from the M2 phenotype to the M1 phenotype. Microbiome-enhanced immunomodulatory therapy is a promising approach in cancer treatment, where alterations in the gut microbiota contribute to improved immune responses against tumours. For example, Shenling Baizhu Decoction (SLBZD) modulates the gut microbiota, which, in turn, influences the TME. This modulation enhances the efficacy of immunotherapies like PD-1 inhibitor by shifting macrophages from an M2 to an M1 phenotype [90]. Additionally, faecal microbiota transplantation (FMT) has been shown to improve responses to immune checkpoint inhibitors in patients with metastatic melanoma, with significant improvements observed in patients who had previously not responded to immunotherapy [91].

Probiotics are also being explored to enhance the efficacy of cancer immunotherapies by promoting a beneficial gut microbiome. This enhancement occurs through mechanisms involving lipopolysaccharides (LPSs) and pathogen-associated molecular patterns (PAMPs) or microbe-associated molecular patterns (MAMPs). These molecules can stimulate the immune system, leading to improved antitumor responses [92,93]. For example, LPS can activate macrophages and dendritic cells, promoting a shift from an immunosuppressive to an immunostimulatory environment [92,93,94,95]. Additionally, gut-located *Lactobacillus reuteri* has been shown to influence macrophage polarisation [96]. For instance, when combined with *Lactobacillus casei*, it can inhibit the proliferation and migration of pancreatic cancer cells and promote M1 macrophage polarisation by suppressing TLR4 [97].

A recent study found that engineered *E. coli phagelysate* (EcPHL) can deliver therapeutic proteins to reprogram macrophages from the M2 to the M1 phenotype, enhancing the immune response against tumours. This reprogramming modifies the TME, increasing the uptake of magnetic nanoparticles by both macrophages and tumour cells. When combined with magnetic nanoparticle hyperthermia, EcPHL significantly suppressed tumour growth in preclinical models, demonstrating its potential to improve the delivery and effectiveness of cancer treatments [98].

Another approach involved engineering *Salmonella* bacteria to produce molecules that inhibit M2 polarisation, promoting M1 conversion [99]. These bacteria selectively targeted tumour-associated macrophages, resulting in significant tumour growth suppression and improved immune responses. Combining these engineered microbial products with existing cancer immunotherapies showed a synergistic effect, reducing tumour size and prolonging survival [99,100].

These examples illustrate the diverse strategies being explored to harness and enhance macrophage activity in cancer immunotherapy.

### Engineering Macrophages

In the context of cancer therapy, macrophages can be engineered using nanotechnology and genetic manipulation to improve their therapeutic efficacy [101]. Innovative approaches to using engineered macrophages for cancer therapy have been explored, including strategies to directly target and eliminate TAMs, reprogram pro-tumour macrophages into anti-tumour phenotypes, and inhibit the recruitment of macrophages into the TME [12,102]. These engineered macrophages not only help in directly attacking tumour cells, but also in reprogramming the TME to support a more robust immune response [89,103]. Additionally, strategies are being developed to ensure the safety and efficacy of these therapies, such as genetic circuits to control therapeutic agent release and safety switches to prevent adverse effects [104].

Engineered macrophages also serve as effective drug delivery vehicles, improving the targeted delivery and efficacy of anticancer therapies [103]. For instance, macrophages can be modified to carry drug nanoparticles directly to tumour sites, enhancing the delivery and effectiveness of anticancer treatments [101]. Advanced strategies, such as macrophage-derived exosomes and macrophage-membrane-coated nanoparticles, further optimise drug delivery and immunotherapy outcomes, highlighting innovative approaches in harnessing macrophages for cancer treatment [105]. Building on these advancements, mannose and glycocholic acid-modified trimethyl chitosan nanoparticles have been developed to deliver SIRPα siRNA and MUC1 pDNA orally. These nanoparticles specifically target macrophages in lymph nodes and tumours, enhancing their immune responses and remodelling the tumour microenvironment. By inhibiting the CD47-SIRPα pathway and promoting a pro-inflammatory M1 phenotype, the engineered macrophages are better equipped to attack cancer cells, ultimately inhibiting the growth and metastasis of triple-negative breast cancer [106].

Antibodies play a crucial role in the engineering of macrophages, particularly in enhancing their ability to target and destroy cancer cells. This involves processes such as antibody-dependent cellular phagocytosis (ADCP), where macrophages use their Fc receptors to bind to the Fc region of antibodies attached to cancer cells, triggering the macrophages to engulf and digest the cancer cells. Additionally, tumours often evade the immune system by expressing proteins that inhibit macrophage activity, and antibodies can block these inhibitory signals, thereby enhancing the macrophages’ ability to attack cancer cells [103]. Using antibodies in combination with engineered macrophages can create a more robust anti-tumour response. Building on this concept, using CpG-free plasmids for non-viral gene delivery into macrophages enables these engineered cells to secrete anti-EGFR single-chain Fv fused with Fc (scFv-Fc). These macrophages effectively target and phagocytise tumour cells expressing EGFR through an antibody-dependent mechanism. When injected around the tumour, they significantly inhibit tumour growth in a xenograft mouse model, showcasing their potential as a powerful strategy for targeted cancer treatment [107].

Expanding on these innovative strategies, a lentiviral vector platform has been developed to selectively engineer liver macrophages, including Kupffer cells and TAMs, to deliver type I interferon (IFNα) to liver metastases. This macrophage engineering significantly delayed the growth of colorectal and pancreatic ductal adenocarcinoma liver metastases in mice by activating TAMs, enhancing MHC-II-restricted antigen presentation, and reducing CD8+ T-cell exhaustion. This approach enhanced the immune response within the tumour microenvironment, increasing the presence of inflammatory TAMs and CD8+ T cells. Additionally, mice that achieved complete responses showed resistance to tumour re-challenge, indicating the induction of memory immune responses [108].

These findings underscore the importance of memory immune responses in achieving long-term cancer remission and highlight the therapeutic potential of ex vivo-generated macrophages. These macrophages, which are primed to different states (e.g., M1 or M2) to enhance their ability to target and destroy cancer cells, have shown promise in clinical applications, outperforming stem cells in specific target diseases. Various genetic modifications to boost the anti-tumour activity of macrophages, including the use of cytokines and other bioactive molecules, are explored [109].

Researchers have explored synthetic biology tools to design bacterial strains capable of sensing and responding to environmental signals for therapeutic purposes. These engineered bacteria can deliver therapeutic payloads, such as proteins or small molecules, directly to target sites within the body, including macrophages. This targeted delivery enhances treatment efficacy by ensuring that therapeutic agents are released precisely where needed. The bacteria can produce and deliver various therapeutic payloads, including cytokines, enzymes, and other bioactive molecules, to modulate immune responses, promote tissue repair, or attack tumour cells. Strategies to improve the safety and efficacy of these engineered bacteria include designing genetic circuits to control payload delivery timing, and location and incorporating safety switches to prevent unintended effects [110,111,112,113,114].

## 4. Bacteria and Macrophages

TAMs are typically derived from circulating monocytes that are recruited to the tumour site, but they can also originate from tissue-resident macrophages. Once in the tumour microenvironment, TAMs can be influenced by various signals, including MAMPs, which can modulate their function and polarisation [115,116]. MAMPs, which include bacterial components like LPS and peptidoglycans, can interact with pattern recognition receptors (PRRs) on macrophages. This interaction can lead to the activation of signalling pathways that influence macrophage behaviour. In the context of cancer, MAMPs can either stimulate an anti-tumour immune response or contribute to an immunosuppressive environment that supports tumour growth and metastasis [8,116]. For instance, certain MAMPs can promote the polarisation of macrophages towards an M1 phenotype, which is associated with anti-tumour activity, while others may drive them towards an M2 phenotype, which supports tissue repair and tumour progression [8,117].

Understanding the dual roles of TAMs and the influence of MAMPs is crucial for developing effective cancer therapies. Targeting TAMs to reprogram them from a pro-tumorigenic to an anti-tumorigenic state is a promising strategy in oncology. This can involve inhibiting macrophage recruitment to tumours, blocking the signals that promote their pro-tumorigenic functions, or enhancing their ability to present antigens and stimulate an effective immune response [118]. These approaches are currently being explored in preclinical and clinical studies, aiming to improve the efficacy of cancer immunotherapies [117,119].

Bacterial interactions with the immune system, pertinent to oncology, lack a detailed synthesis in existing literature; thus, documenting these dynamics is crucial for laying the groundwork to strategically exploit such mechanisms for therapeutic benefit [120]. The following sections presents a summary of the recent progress in the field of bacterial reprogramming of TAM, highlighting its potential in the development of cancer treatments.

### Natural Bacterial Immunomodulation

Bacteria naturally stimulate proinflammatory (anti-tumour) immune responses through mechanisms involving MAMPs like LPSs, peptidoglycans, lipoteichoic acid, flagellin, and nucleic acids. These MAMPs activate innate immunity by binding to PRRs, such as Toll-like, NOD-like, RIG1-like, C-type lectin, and AIM2-like receptors, on dendritic cells, macrophages, monocytes, and B lymphocytes. These PRRs are germline encoded receptors in innate immune cells, mainly by antigen-presenting cells (APCs), that act as broad specificity antigen recognition sites [118].

PRR activation in APCs initiates adaptive cognate interactions and triggers a cascade of signalling events, leading to epigenetic and metabolic rewiring that modulates innate immune cell function. Recent evidence shows that PRR activation by bacterial MAMPs activates pro-survival and proliferation pathways, including the IL-6/NF-κB/STAT3 epigenetic feedback loop, the MAPK proliferation pathway, and the IL-23/IL-17 pro-carcinogenic pathway [121,122,123].

These complex signalling pathways highlight how bacterial MAMPs not only initiate immune responses, but also modulate them through various mechanisms. LPS promotes the DC maturation and M1 polarisation of macrophages via TLR4-mediated signalling pathways [124]. Flagellin activates immunoinflammatory responses through TLR5 recognition on antigen-presenting cells [125]. Moreover, specific TLR2 agonists from Gram-positive bacteria modulate the phenotypes and functions of immune cells, initiating anti-cancer responses [124]. Cytosine–guanine oligodeoxynucleotide (CpG ODN) motifs in bacterial and synthetic DNA serve as potent TLR9 agonists, used as vaccine adjuvants to promote antigen presentation and elicit anti-cancer T cell responses [126,127,128].

PRR activation in innate effector cells triggers the expression of cell-specific inflammatory and effector cytokines, which modulate the intensity of the immune response [129,130]. In macrophages, bacterial MAMPs trigger pro-inflammatory immune responses through their interaction with PRRs, which play a crucial role in the innate immune response [131]. This interaction activates signalling pathways that drive macrophage polarisation [132]. The secretion of type 1 cytokines (IFN-γ, TNF-α) induces macrophage polarisation towards the pro-inflammatory M1 phenotype, while type 2 cytokines (IL-4, IL-10) promote the anti-inflammatory M2 phenotype [130,133].

**Figure 2:**
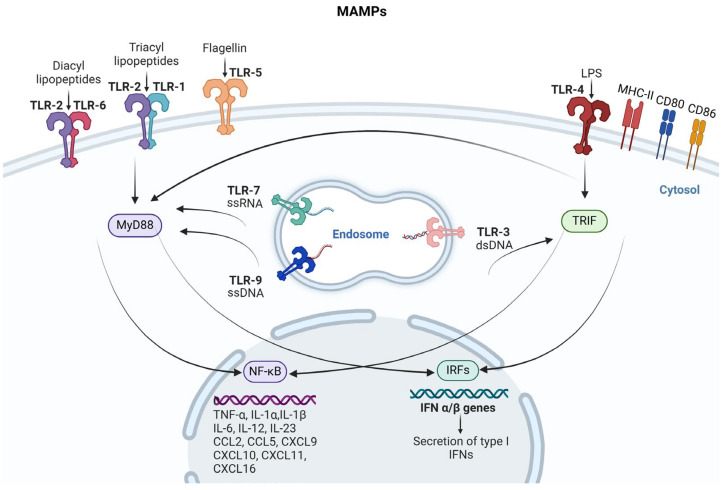
Activation of cytokine cascade by MAMPs in macrophages. Upon interaction with microbe-associated molecular patterns (MAMPs), macrophages initiate a robust pro-inflammatory immune response through the engagement of pattern recognition receptors (PRRs) such as Toll-like receptors (TLRs). This interaction activates key adaptor proteins, including MyD88 and TRIF, which subsequently trigger downstream signalling pathways. MyD88 predominantly activates NF-κB pathway, leading to the transcription of pro-inflammatory cytokines. TRIF, on the other hand, activates IRFs, promoting the production of type I interferons. These signalling events drive macrophage polarisation, with M1 polarisation characterised by the production of pro-inflammatory cytokines and increased expression of surface markers such as MHC-II, CD80, and CD86. This cascade ensures an effective immune response, amplifying cytokine production and recruiting additional immune cells to the site of infection.

Macrophages play a crucial role in natural bacterial immunomodulation. These innate immune cells are the first responders to infection, recognising and engulfing pathogens. Upon encountering bacterial MAMPs, macrophages utilise PRRs to detect these molecular patterns, triggering a cascade of signalling pathways. This leads to the activation of adaptor proteins like MyD88 and TRIF, which, in turn, activate transcription factors such as NF-κB and IRFs. The result is the production and release of pro-inflammatory cytokines, which are essential for initiating and sustaining the immune response [133,134]. Figure 2 illustrates the interaction between MAMPs and macrophages, highlighting the subsequent activation of the cytokine cascade.

These findings underscore the multifaceted role of bacterial MAMPs in shaping immune responses. By engaging various PRRs, these microbial components not only initiate but also modulate immune cell functions, leading to diverse outcomes ranging from proinflammatory responses to adaptive immunity. The intricate interplay between these pathways highlights the potential of leveraging bacterial MAMPs in therapeutic strategies, particularly in the context of cancer immunotherapy. Understanding these mechanisms further opens avenues for developing targeted interventions that harness the body’s innate and adaptive immune systems to combat malignancies.

The upcoming section explores contemporary strategies related to using bacteria as carriers for immunotherapy delivery, focusing on macrophages.

## 5. Bacteria and Cancer

In the past 150 years, bacteria have been associated with a positive effect tumour therapy in clinical practice [134]. Beyond the gut microbiota, resident bacteria within local tissues have been implicated in various phases of cancer progression [120]. Recent studies have indicated a renewed scientific interest in the oncobiome, particularly focusing on the contributory role of bacterial agents in modulating the efficacy of anticancer therapies and their potential to alter therapeutic outcomes [135].

The association between host immun ity and the microbiome in modulating responses to different cancer therapeutics, including metabolism, the immune response, and the translocation of microbiome constituents, presents opportunities for the development of novel strategies to improve clinical outcomes [136]. The active function of the microbiota in regulating immune processes both systemically and in the TME have promoted investigations on related impact on cancer immunotherapies, revealing associations (both positive and negative) between microbiota composition and treatment outcomes [120,137], making it an attractive possibility to combine live bacteria with other immunotherapeutic approaches [138,139,140].

### 5.1. Bacterial Tumour Targeting

Bacteria possess unique properties that allow them to selectively target tumours following systemic administration [141]. Their surface structures or metabolites can activate the immune system, leading to anti-tumour effects. As a result, bacteria are well-suited to serve as therapeutic agents themselves or as DNA or protein delivery vehicles in tumour immunotherapy [142].

The selective replication of bacteria within tumour sites involves several key factors that distinguish malignant tissue from healthy tissue. The irregular blood vessels within malignant tissues enable bacteria to lodge in tumour tissue, whereas TNF-α production induced by certain bacteria has been postulated to promote local haemorrhage and retain bacteria in these irregular blood vessels [143,144,145]. The conditions within the TME then support bacterial replication. The irregular and chaotic blood vessels result in poor oxygen and nutrient diffusion. As a consequence, regions of hypoxia and necrosis arise in tumours [127,128], where many facultatively anaerobic bacteria can thrive due to low oxygen levels and nutrients released by necrotic cancer cells [146,147]. Additionally, the local immunosuppressive environment prevents early bacterial clearance [148]. Research models have shown this tumour-selective replication to be tumour type agnostic, while more recently, a growing body of literature is reporting the presence of a range of bacterial types within patient tumour tissue [118,148,149,150].

The TME in a broad spectrum of human cancers is increasingly being proposed to feature unique microbiota, potentially playing a role in tumour progression and the development of resistance to anticancer therapies [151,152]. The dynamic changes in the TME, particularly the induction of M1 macrophage polarisation, play a pivotal role in promoting an anti-cancer environment. As a result, the intratumoural microbiota has potential to be a valuable complementary agent that may enhance the effectiveness of therapies aimed at increasing the accumulation of intratumoural IFN-γ and CD8+ T-cells, thus highlighting its significant potential to strengthen the anti-tumour immune response [153].

### 5.2. Utilising Bacteria as Nucleic Acid and Protein Delivery Vehicles

Tumour-targeting bacteria are ideal biological gene vectors with natural tumour specificity, capable of homing to tumours and replicating locally to high levels when systemically administered [154,155]. Advances in genetic engineering have garnered significant interest due to their dual capabilities: firstly, the targeted localisation within tumour tissues, facilitating the delivery of therapeutic agents to regions beyond the reach of conventional treatments; and secondly, their expansive gene packaging potential, which enables the expression of substantial and diverse therapeutic proteins [111,141,156].

Bacteria have emerged as innovative carriers for tumour therapy, owing to their adaptability for customisation and their natural inclination to infiltrate tumour environments [157]. These bacterial vectors can be readily programmed through cutting-edge DNA synthesis, intricate genetic engineering, and biosensor technology. This enables the creation of microorganisms with elaborate functionalities that can produce therapeutic agents on-site, such as regulating the immune response within the tumour [144,158]. Bacteria engineered to carry therapeutic agents can strategically deliver these treatments within solid tumours, reshaping the immune landscape. Recent studies demonstrated that these modified bacteria can persist within tumours, ensuring a consistent and long-lasting spread of the therapies in both human and mouse tumour models [159,160,161].

Antitumor therapy involving bacterial genetic manipulation, tumour targeting specificity, and immune system modulation holds immense promise for clinical applications. However, the side effects and limited efficacy of current treatments hinder their widespread use in biomedicine. To address this, researchers have explored engineered bacteria that can selectively target tumours while minimising toxicity to normal organs [162].

Additionally, the inherent targeting attributes and anticancer properties of bacterial elements, such as vesicles, spores, toxins, metabolites, and various bioactive compounds, are leveraged [163]. These elements act as transport mechanisms for diverse biomolecules, including proteins, lipids, DNA, mRNA, and microRNAs [164]. Upon delivery to macrophages, these molecules orchestrate the modulation of numerous signalling cascades, culminating in the induction of macrophage polarisation towards an M2 phenotype [165].

## 6. Bacterial-Based Programming of Macrophages

**Figure 3:**
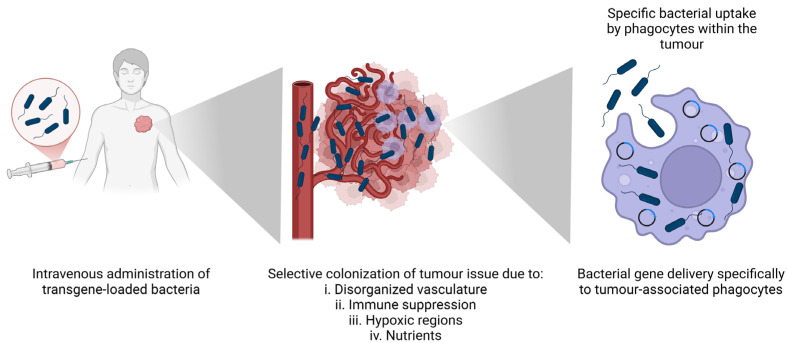
In situ engineering of TAMs by bacteria. Genetically engineered bacteria are administered intravenously. These genetically engineered bacteria are attracted to the tumour microenvironment due to its hypoxic conditions, nutrient-rich environment, immunosuppressive properties, and aberrant vasculature. Upon reaching the tumour site, the bacteria are phagocytosed by tumour-associated macrophages, where they release plasmids that subsequently express therapeutic agents to modulate the tumour microenvironment and inhibit tumour growth.

The gene transfection capability of bacteria is typically restricted to invasive bacteria, with the unique exception of phagocytic cells. Non-invasive (and therefore safer) bacteria possess the ability to ‘passively’ transfect macrophages. This has been known for some time in the context of use of bacteria in delivery of DNA vaccines, for antigen delivery to APC [166]. Our group demonstrated the potential to selectively transfect TAMs using non-invasive bacteria [111]. These bacteria are preferentially taken up by phagocytic cells, ensuring targeted and efficient DNA delivery to TAMs. The efficacy of this method has been validated both in vitro with human monocyte cell lines and in vivo in mouse models using a reporter gene strategy. Solid tumour models demonstrated the ability of bacteria to transfect intratumoural TAM following systemic intravenous administration, while peritoneal ascites models demonstrated successful transfection of ‘suspension’ macrophages following intraperitoneal injection. Notably, the bacteria not only facilitated gene delivery (as evidenced by the host cell production of reporter protein), but also enhanced the recruitment of additional phagocytic cells to the tumour microenvironment. This strategy holds significant promise for developing targeted therapies aimed at modulating the TME through the selective transfection of TAMs and may be applicable to other pathologies involving phagocytic cells [111]. Figure 3 illustrates the process of in situ engineering of TAMs by bacteria, highlighting their journey and function within the tumour microenvironment. 

### Other Re-Polarisation Strategies Employing Bacteria

Advances in genetic engineering have enabled the modification of bacteria to directly or indirectly induce repolarisation from M2 to M1 phenotypes. Both the polarisation induced by live bacteria and the immunomodulatory effects of bacterial components contribute to this shift, enhancing the immunotherapeutic potential in oncology [167].

Among the various strategies, those that have shown the most promise in clinical settings involve the use of genetically engineered bacteria and bacterial components. For instance, clinical trials have demonstrated that reprogramming macrophages using HDAC6 inhibitors can significantly increase the M1/M2 ratio, leading to enhanced antitumor immune responses in melanoma patients [168]. Additionally, the use of TAM-reprogramming agents in over 700 clinical trials has highlighted the potential of these therapies to transform the tumour microenvironment (TME) and improve patient outcomes [169].

Overall, the integration of genetically engineered bacteria and bacterial components into cancer immunotherapy marks a significant leap forward in oncology. These innovative strategies have demonstrated remarkable potential in transforming the TME by reprogramming macrophages from an immunosuppressive M2 phenotype to a pro-inflammatory M1 phenotype. This reprogramming enhances the body’s immune response against tumours. The promising outcomes from these diverse methods highlight the potential of microbial elements to develop more effective and targeted cancer treatments, offering new hope in the ongoing battle against cancer. Table 1 summarises various bacterial-based strategies for macrophage repolarisation, highlighting the key findings and references for each approach.

While a range of therapeutic approaches that facilitate the transition from M2 to M1 macrophages have demonstrated efficacy in preclinical cancer treatment models, it is important to note that there are currently no clinical studies specifically using bacteria to reprogram macrophages from M2 to M1 phenotypes. Most of the research in this area has been conducted in vitro or in murine models. Therefore, the promising outcomes observed in these preclinical studies highlight the potential of these strategies, but further clinical research is needed to validate their effectiveness in human patients.

Additionally, recent studies have demonstrated that non-pathogenic, genetically engineered strains of bacteria can be used safely in cancer treatment. These bacteria are designed to selectively target tumour cells while minimising the risk to healthy tissues. For instance, research has shown that these engineered bacteria can be controlled to release therapeutic agents specifically within the tumour microenvironment, significantly reducing systemic toxicity [113]. Moreover, advancements in genetic engineering have made these bacterial strains at least 100 times safer than previous iterations, with mechanisms in place to prevent premature clearance and unintended invasion of healthy cells [188].

However, the use of engineered bacteria to reprogram TAMs presents inherent safety concerns, including the risk of unintended immune responses and infections, necessitating rigorous safety assessments and long-term monitoring [112]. Additionally, the scalability of using engineered bacteria for widespread clinical application remains a significant challenge, as current studies are often limited to small-scale experiments [189]. Translating these findings from preclinical models to clinical settings poses several challenges due to variability in human immune responses and tumour microenvironments [161]. Future research should focus on developing advanced safety protocols, optimising bacterial strains for enhanced specificity and efficacy, and investing in scalable production techniques [190]. Comprehensive clinical trials are essential to evaluate the safety and efficacy of bacterial reprogramming in diverse patient cohorts. Additionally, investigating the integration of bacterial reprogramming with existing cancer therapies may enhance overall therapeutic outcomes [191]. Addressing these limitations and following the outlined roadmap could significantly advance the field of cancer therapy and improve patient outcomes through innovative approaches to TAM reprogramming [192].

Clinical trials are underway to further assess the safety and efficacy of these treatments, with early results indicating a favourable safety profile [191]. However, the use of live bacteria injections in cancer therapy, while promising, presents several potential adverse effects that must be carefully considered. These include the risk of unintended immune responses, such as excessive inflammation or autoimmunity, and the possibility of infections, particularly in immunocompromised patients [193]. Additionally, there is a concern about the potential for bacterial persistence or dissemination beyond the target site, which could lead to systemic infections [194]. To mitigate these risks, rigorous safety assessments, including preclinical studies and controlled clinical trials, are essential [195]. These studies should focus on optimising bacterial strains to enhance their safety profile and developing protocols for monitoring and managing adverse effects [113]. Addressing these safety concerns is crucial for the successful clinical translation of bacterial reprogramming therapies [196].

## 7. Conclusions

In conclusion, remarkable advances in cancer research have led to the development of innovative drugs and therapies, significantly impacting the role of macrophages in cancer progression and treatment. These advances are crucial because macrophages, depending on their activation state, can either suppress immune responses or enhance antitumor activity, making them pivotal in cancer therapy. TAMs, which are a significant component of the tumour microenvironment, often correlate with poor prognosis across various cancer types due to their high infiltration and immunosuppressive characteristics. This correlation underscores the importance of targeting TAMs in cancer treatment. The latest strategies to switch macrophage polarisation focus on re-educating TAMs by targeting molecular pathways that regulate macrophage polarisation, such as cytokines, chemokines, transcription factors, receptors, and miRNAs. These strategies also include inhibiting the recruitment of M2-like TAMs to the tumour microenvironment, enhancing phagocytosis by TAMs to clear cancer cells, and reprogramming TAMs to remodel their anti-tumour capacity, potentially converting them into M1-like macrophages with pro-inflammatory and tumour-suppressing functions. Understanding the complex interactions between tumour cells, the tumour microenvironment, and infiltrating immune cells like macrophages is crucial for developing these new therapeutic strategies. By manipulating the polarisation of macrophages, it is possible to create a more hostile environment for cancer cells, thereby inhibiting tumour progression and supporting the body’s natural defences against cancer.

Engineered bacteria represent a novel immunotherapeutic strategy that enables targeted immunoregulation by depleting TAMs, inhibiting new TAM differentiation, and re-educating them for enhanced cancer cell phagocytosis. This approach is promising because bacterial therapy can modulate macrophage polarisation in cancer, shifting macrophages from a pro-tumour M2 phenotype to an anti-tumour M1 phenotype, thereby improving therapeutic outcomes. Bioengineering technology has revealed the vast potential of engineered bacteria in immunotherapy. These bacteria serve as immunogenic vectors, cytokine producers, antibody generators, and converters of tumour waste into nutrients. Their participation opens innovative opportunities for combination immunotherapy, allowing for flexible switching between immunoregulatory enhancement, increased immunogenic cell death, and direct immunotherapeutic effects. Ultimately, bacterial strategies hold promise for reshaping the tumour microenvironment by influencing macrophage polarisation. As research advances, these approaches may contribute to more effective cancer therapies. Leveraging the essentially unlimited gene packaging capacity and neoplasm specificity of modified oncotropic bacteria, combined with adjunct immunotherapeutic modalities, is anticipated to emerge as a successful therapeutic strategy imminently. The integration of bacterial therapy in targeting TAMs offers transformative potential for more effective and personalised cancer treatments, highlighting the importance of continued research and development in this innovative field.

## Figures and Tables

**Table 1:** Bacterial-based strategies for macrophage repolarisation.

Repolarisation Strategy	Bacteria	Key Findings	Study Type	Reference
Live Bacteria	*Escherichia coli*	Mediates DNA delivery to tumour-associated phagocytic cells, enhances gene expression within tumours	*in vitro*	[145]
	*Salmonella VNP-PD1nb*	Reactivates TME, increases immune cell infiltration, shifts TAMs to M1 phenotype, activates CD8+ T-cells	murine *in vivo*	[100]
	*Salmonella* Typhimurium VNP20009 (VNP-IFNb)	Enhances PD-1 blockade therapy, reduces PD-1 and VEGF levels, decreases M2 macrophages, boosts T-cell activity	murine *in vivo*	[170]
	*Cutibacterium acnes*	Infiltrates TME, attracts M2 macrophages, impedes tumour progression	murine *in vivo*	[171]
	*Enterococcus faecalis*	Enhances macrophage viability, induces atypical M1-like polarisation, persists despite M2-inducing factors	*in vitro*	[172]
	*Salmmonella*	Recruit’s neutrophils and macrophages, activates dendritic cells, inhibits melanoma progression	murine *in vivo*	[173]
	*Escherichia coli* MG1655	Delivers tumour necrosis factor alpha (TNFα) specifically within tumours, inhibits tumour growth, induces pro-inflammatory cytokine production	murine *in vivo*	[111]
	*Salmonella* Typhimurium	Induces M1 macrophage polarisation, enhances anti-tumour immune response	murine *in vivo*	[173]
	*Bifidobacterium longum*	Promotes M1 macrophage polarisation, reduces tumour growth	murine *in vivo*	[112]
Bacterial component	*Lactobacillus casei (ferrichrome)*	Enhances anti-PD-L1 treatment, modulates TAM polarisation, increases CD8+ T-cells, reduces MDSCs	murine *in vivo*	[174]
	*Escherichia coli* Nissle *1917 (Ovalbumin and α-PD-1)*	Promotes dendritic cell maturation, activates cytotoxic T lymphocytes, converts TAMs to M1 phenotype	murine *in vivo*	[167]
	*Bacterial* exopolysaccharides (EPS)	Inhibits cell growth, initiates programmed cell death, reduces inflammation	*in vitro*	[175,176]
	Nanovesicles (NVs) with Cas9-sgRNA and CpG-rich DNA	Remodels TME, stabilises M1-like phenotype in TAMs, inhibits tumour growth	murine *in vivo*	[177]
	Epidermal growth factor receptor (EGFR) of platelet	Enhances macrophage immune function, induces M1 macrophage polarisation, improves bacterial clearance	*in vitro*	[178]
	Bacterial lipopolysaccharides (LPS)	Triggers M1 macrophage polarisation, enhances immune response	*in vitro*	[179]
	Poly(lactic-co-glycolic acid) (PLGA)*-*R848 (PR848) on Escherichia coli (Ec-PR848)	Targets drug delivery, polarises M2 macrophages to M1, enhances anti-tumour immune response	murine *in vivo*	[180]
Bacterial-derived nanoparticles	Gold nanoparticles (GNPs)	Activates photothermal properties, improves immune cell infiltration, enhances checkpoint blockade immunotherapy	murine *in vivo*	[181]
	DOX@TA@ECN	Combines chemotherapy and immunotherapy, regulates ECN numbers, induces immune-mediated anti-tumour responses	murine *in vivo*	[182]
	*Veillonella atypica* (VA-SAM@BTO)	Targets colorectal cancer, catalyses immunogenic cell death, disrupts immunosuppressive microenvironment, enhances dendritic cell maturation, polarises macrophages toward the M1 phenotype	murine *in vivo*	[183]
	*Bifidobacterium bifidum* B.b@QDs	Mediates TAM polarisation, enhances immune response, inhibits tumour growth	murine *in vivo*	[184]
	Outer Membrane Vesicle (OMVs) modified with FeIII-tannic acid (*FeIII-TA)*	Reduces systemic toxicity, improves tumour targeting, shifts TAMs to M1 phenotype, triggers strong anti-tumour immune response	murine *in vivo*	[185]
	OMV-CD47nb (PEG/Se@OMV-CD47nb)	Promotes engulfment of tumour cells, encourages M1 polarisation, boosts T-cell-driven antitumor immunity	murine *in vivo*	[186]
	Interferon-gamma (IFN-γ) bacterial biofilmvesicle (BBV) (IFN-γBBV)	Reprograms TAMs to M1 phenotype, enhances CTL response, inhibits tumour growth	murine *in vivo*	[187]

Abbreviations: TME: tumour microenvironment; TAMs: tumour-associated macrophages; MDSCs: myeloid-derived suppressor cells; CTL: cytotoxic T lymphocytes; PD-1: programmed cell death protein 1; VEGF: vascular endothelial growth factor; TNFα: tumour necrosis factor alpha; PLGA: poly(lactic-co-glycolic acid); GNPs: gold nanoparticles; DOX: doxorubicin; TA: tannic acid; ECN: *Escherichia coli* Nissle; QDs: quantum dots; OMVs: outer-membrane vesicles; IFN-γ: interferon-gamma.
